# Using a commodity high-definition television for collaborative structural biology

**DOI:** 10.1107/S160057671400939X

**Published:** 2014-05-29

**Authors:** Ragothaman Yennamalli, Raj Arangarasan, Aaron Bryden, Michael Gleicher, George N. Phillips

**Affiliations:** a Department of Biochemistry and Cell Biology, Rice University, 6100 Main Street, Houston, Texas 77005, USA; b The Raj Organization, Henderson, Nevada 89074, USA; c Department of Computer Sciences, University of Wisconsin–Madison, Wisconsin 53706, USA; d Praxik LLC, 2701 Kent Avenue, Suite 130, Ames, Iowa 50014, USA

**Keywords:** collaborative structural biology, protein structure visualization

## Abstract

Visualization of protein structures using stereoscopic systems is frequently needed by structural biologists working to understand a protein’s structure–function relationships. Often several scientists are working as a team and need simultaneous interaction with each other and the graphics representations. Most existing molecular visualization tools support single-user tasks, which are not suitable for a collaborative group. Expensive caves, domes or geowalls have been developed, but the availability and low cost of high-definition televisions (HDTVs) and game controllers in the commodity entertainment market provide an economically attractive option to achieve a collaborative environment. This paper describes a low-cost environment, using standard consumer game controllers and commercially available stereoscopic HDTV monitors with appropriate signal converters for structural biology collaborations employing existing binary distributions of commonly used software packages like *Coot*, *PyMOL*, *Chimera*, *VMD*, *O*, *Olex2* and others.

## Abstract 2

A method exploiting off-the-rack television and game controllers for use by crystallographers and their collaborators for enhanced experience of co-located discussions is presented.

## 1. Introduction   

Unlike the flattened visualization of proteins, stereoscopic visualization not only is visually pleasing but helps the viewer to understand the complexity of the molecule. While non-stereo images may provide a range of depth cues, such as lighting and occlusion, stereo displays have a historical popularity in molecular visualization. There has been an interest in seeing images that ‘float without a frame’ since the 1800s (Lee, 2013 ▶). The field of structural biology is inherently multi-disciplinary, as it includes both those who understand crystallography and molecular geometry and others who are interested in how proteins perform within biological systems. Structural biologists frequently visualize proteins with their collaborators in group settings called co-located collaborations. In these discussions, they discuss the protein structure and its other properties.

Co-located collaborations are essential in structural biology. Cross-disciplinary teams need to work together, referring to and studying visualizations of the molecules’ shapes and properties. In such teams, participants discuss proteins in context with other experimental determinations of their functions, which are usually known collectively only by a specific set of investigators. This is especially true in the area of structural genomics, where the person who solves the protein structure may have less knowledge of functional experiments than others. This disconnect hinders the effectiveness of collaborations. Discussions between scientists about both the structure and the functional experiments make collaboration productive by allowing group discussions, as structural biologists utilize a variety of abstract representations of these molecules and visualization options to best understand the molecule they are examining. This paper describes a method that structural biologists can utilize for co-located collaborations in a more effective format.

Molecular visualization has a rich toolbox of visualization and analysis techniques, from the earliest available tool developed by Cyrus Levnithal (1966 ▶) to a wide range of tools available today. Structural biologists have used a wide variety of representations to visualize proteins and their dynamics, such as spheres and stick bonds, ribbon representations (Lesk & Hardman, 1982 ▶), solvent excluded surfaces (Sanner *et al.*, 1995 ▶), ambient occlusion shading (Tarini *et al.*, 2006 ▶), abstracted molecular surfaces (Cipriano & Gleicher, 2007 ▶), and molecular dynamics movies (Phillips *et al.*, 2005 ▶), to name a few. O’Donoghue *et al.* (2010 ▶) describe these techniques in detail. There are several popular molecular visualization packages, including *VMD* (Humphrey *et al.*, 1996 ▶), *PyMOL* (http://www.pymol.org/) and *Chimera* (Pettersen *et al.*, 2004 ▶). Additionally, *Coot* (Emsley *et al.*, 2010 ▶), *O* (Jones *et al.*, 1991 ▶) and *Olex2* for small-molecule crystallography (Dolomanov *et al.*, 2009 ▶) are popular tools with crystallographers for visualizing the electron density map and for manipulating structures.

## 2. Co-located collaborations   

Co-located collaborations refer to collaborative work settings that take place in the same physical location. Structural biology collaborators can engage in group discussions of protein structure. In our experience, these discussions are more effective when facilitated by a large, vertical, stereo display. These discussions typically revolve around specific properties of amino acid side chains or surfaces of a protein molecule and are usually relevant to some other domain of study such as enzymology or molecular recognition. For these discussions to be effective, they need to capitalize on one of the multitude of visualization and analysis options that are widely used in single-user desktop molecular visualization software. Also the collaborators need to have expertise in using the software. A variety of display and interaction paradigms for co-located collaboration currently exist, including immersive environments such as tabletop displays, large tiled displays, domes and multiple co-located displays.

Bryden *et al.* (2011 ▶) explored issues in adapting traditional mol­ecular visualization software for co-located use. They examined the needs in co-located interactions, designed interaction techniques that supported these needs and validated the novel tools in a study on human subjects. However, one of their findings was that many of the benefits of their solution can be obtained without necessarily using all of the components. In this paper, we provide a practical realization of such a partial design.

### 2.1. Limitations of existing methods: usage   

In co-located collaborations such as those that use displays like the VizBox stereo projection system or a large tiled display, the limitation of crowding to a smaller screen is overcome. However, when the conversation involves multiple people, a number of new issues arise (Bryden *et al.*, 2011 ▶). First, in some systems, only a single person can ‘drive’ the visualization (*i.e.* control the system, including manipulating the viewpoint). Switching ‘drivers’ is time consuming because the driver needs to sit at the console. Therefore, the common usage paradigm is to have a ‘designated driver’, relegating other participants to take a ‘back seat’. Here, the participants communicate by recommending viewpoint changes to the driver, offering a less participatory experience. Because of the limitations of the mouse pointer, it is quite common for participants to attempt to point out parts of the molecule with their hands. While in some discussions, this can work quite well, the use of stereo viewing causes much confusion when participants use their hands for pointing because of parallax issues. Pointing by fingers is tempting but ineffective because each person has a different viewpoint, plus it obscures the view. In such displays, there is no explicit support for switching between multiple viewpoints and selection sets, and participants spend considerable time recreating previous configurations. For example, if a structure contained an important binding site, it might be desirable to look at the binding site directly (*e.g.* from the top view) and also view it from another angle (*e.g.* side view) to see how the surrounding geometry affects it. Similarly, when examining the dynamics of conformational change in a molecule, it may be desirable to switch between an overview of the whole molecule and a close-up view of the active site. These options are not employed in the current non-co-located collaborations. Additionally, it is difficult for the ‘designated driver’ to change the view effectively or move the pointer to an appropriate position because of the configuration of the space.

### 2.2. Limitations of existing methods: hardware   

Currently, practical issues challenge the use of stereo visualization on consumer displays. While emerging video standards are capable of supporting high-resolution stereo, not all devices support the most current standards. Full support of standards is particularly problematic when we seek to use lower-cost devices.

### 2.3. Commodity TVs and explanation of various stereo modes   

There are many reasons that make commercially available stereoscopic high-definition televisions (HDTVs) an attractive option for co-located discussions. Commodity stereoscopic HDTVs that are stereo capable have several modes of providing the left- and right-eyed views necessary for creating the stereo effect. Either active stereo glasses (LCD shutters) are used to separate the two signals in time, or passive glasses can use polarized light to allow filtering of the left and right views with polarizing filters on the glasses. The ways the signals are processed by the TV include the single signal being interleaved with analog and digital signals or using half of the vertical or horizontal resolution (or a checkerboard pattern) of pixels in an alternating fashion. The TV circuitry alternately either displays the signal in sync with the LCD shutters or delivers the light though appropriate polarizers built into the screen. With passive displays (not in passive projection displays) there is diminution of resolution due to the alternating use of pixels from the video signal.

The application graphics software must be designed to deliver one of these formats (interleaved or side by side). A better alternative is to use the HDMI 1.4a HDTV standard, which uses alternating full-resolution images pre-encoded to sync with the TV’s control of the LCD shutters. This provides the full 1080p resolution of the TV signal. This can be achieved by using some inexpensive commercial broadcast electronics and a professional grade graphics card (in this case from NVIDIA or AMD) in a Windows PC. If the applications support quad-buffered stereo, then the large-format HDTV can be used without recompiling or changing the application software.

### 2.4. Using Xbox controllers for co-located collaboration   

Among the various input devices currently available (such as Wii, Xbox and multiple mice), we realized that the input device type with the most prior use in group settings at the consumer level is the video game controller, such as Xbox, owing to its portability, ease of use in a single-display group setting, ability to perform view manipulation and pointing as core metaphors, and having enough buttons to support additional tasks (Bryden *et al.*, 2011 ▶). Also, the availability of software that can interface the video game controller with multiple functionalities (such as keyboard functions) makes it an attractive option for a multi-user setup as seen in a co-located collaboration.

In this paper, we describe our experience developing a functional collaborative molecular visualization tool using consumer-level stereoscopic HDTV monitors together with standard consumer game controllers for stereoscopic enabled OpenGL applications. Our goal is to develop a system to support a collaborative visualization environment that is inexpensive and effective and does not require any changes to the existing software applications. Our premise is that group collaboration is best supported by simple systems specifically designed to address the type of work being done. Although current molecular visualization tools do support a variety of displays to be used in co-located collaborations, they are designed to support single-user tasks and so are not necessarily well suited for collaborative work.

## 3. Materials and setup   

The stereoscopic HDTV visualization setup was implemented in a Windows 7 64 bit (Ultimate) operating system equipped with 16 GB RAM and an Intel Xeon processor (64 bit). The low-end consumer-grade graphics card usually does not support an HDMI 1.4a stereoscopic output directly from OpenGL applications. At the time we purchased the components, there were few available off-the-shelf options that supported the HDMI 1.4a stereoscopic conversion from SDI signals. Thus, we used the quad-buffer-enabled graphics cards and an NVIDIA Quadro 6000 SDI card instead of the AMD card.

**Figure 1:**
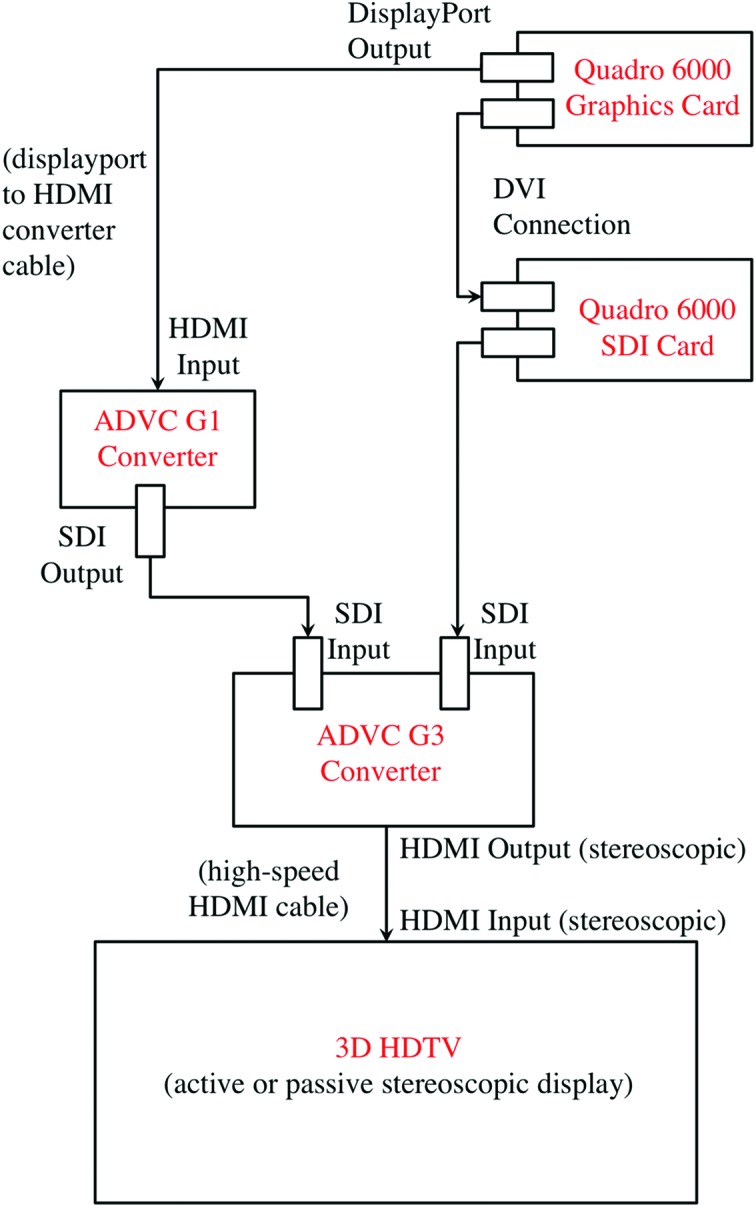
Schematic diagram of a stereoscopic HDTV setup for collaborative visualization. The diagram shows the output from the Quadro 6000 Graphics and SDI card converted by the ADVC G1 and G3 converter to an HDMI output, which is then displayed on a stereoscopic HDTV *via* quad-buffered stereoscopic view.

External converters were used to create real-time HDMI 1.4a stereoscopic output video signals at hardware level, since the graphics card does not provide the native HDMI 1.4a full HD stereoscopic output for OpenGL applications. The converters, ADVC G1 (HDMI/DVI to SDI converter) and ADVC G3 (2X SDI to HDMI1.4a converter), were obtained from Grassvalley Inc. The appropriate driver, NVIDIA Quadro SDI display driver, was downloaded from http://nvidia.com. Apart from the above high-speed HDMI cables (Displayport to HDMI, HDMI to HDMI), SDI cables and active stereoscopic glasses completed the setup. Fig. 1 ▶ shows a schematic diagram of the complete setup.

For implementing the Xbox controllers, we chose Pygame version 1.9.2 and a modified OpenGL Xbox controller driver. We were able to use a general-purpose device-mapping program (*Xpadder*; http://www.xpadder.com) to map the mouse and keyboard functionalities for the game controllers.

## 4. Results and discussion   

Structural biologists need visualization methods that enable a multiple-user interface. Various display paradigms exist, such as immersive environments, tabletop displays, large tiled displays, domes and geowalls (Akkiraju *et al.*, 1996 ▶; Achalakul *et al.*, 2004 ▶; Li *et al.*, 2003 ▶; Maxfield *et al.*, 1998 ▶; Forlines & Lilien, 2008 ▶; Tate *et al.*, 2001 ▶). From the user’s point of view, because there may be different points of view for looking at the same molecule, moving between viewpoints of each participant is common, and considerable time is spent recreating previous views. For example, with a projection display system, participants often use pointing gestures to refer to places on molecules. These gestures are done either with their hands or with the mouse pointer if they have access. Depending on the resolution of the projector(s) used and their alignment, the clarity of the final image can range from acceptable to poor. With a large tiled display, the seams that join these displays create a grid-like view of the projected image that can obscure an effective visualization.

Here, we describe a method of visualization that uses HDTV and has a multi-user interface. Fig. 1 ▶ shows a schematic implementation of a commercially available HDTV with the quad-buffer-enabled graphics cards and output sent *via* HDMI1.4a cables. The multiple participants in a co-located discussion require multiple-user input devices for effective communication. These input devices should be able to support view manipulation and pointing as core metaphors and have enough buttons to support additional tasks. To meet this requirement, we used Xbox 360 controllers because they are the most comfortable among the available dual stick controllers; also they support multiple wireless controllers simultaneously. By using these controllers, participants can clearly communicate which part of the molecule they are referring to and furthermore effectively select residues, atoms and chains and rotate objects. Instead of using one’s hands in a single stereo display, we give each user a pointer inside the system.

Providing each user their own input device has significant advantages, but it also has the potential for users’ actions getting confused, when they originate from two or more users at the same time. So, a floor control model is implemented that attempts to prevent conflicting access without requiring explicit coordination. The model uses two states, so that in one state anyone may take control by beginning an action such as viewpoint control. Once a user has taken control, they have exclusive control while they complete their action, and, for a brief period afterwards, this position allows them to start a new action (to accommodate pauses). In doing so, the floor control helps prevent confusion by allowing each user to know when they are in control or another user is in control. To implement our design we created a plugin called *CollabMOL* to the existing *PyMOL* molecular visualization software (https://graphics.cs.wisc.edu/WP/blog/2011/09/02/collabmol-pymol-plugin-info/).

### 4.1. *Xpadder* usage   

We also implemented keyboard/mouse-mapping functions to the Xbox controllers. *Xpadder* software simulates the keyboard and mouse functionalities to gamepad controllers, such as Xbox controllers. Although *Xpadder* is written with the aim of using Xbox controllers for games such as those with no gamepad support, we found that many of the routinely used mouse and keyboard commands in *PyMOL*, *VMD* and *Coot* can be executed using the Xbox controller. *Xpadder* is quick to set up, cost effective and does not require changes to the existing applications.

**Figure 2:**
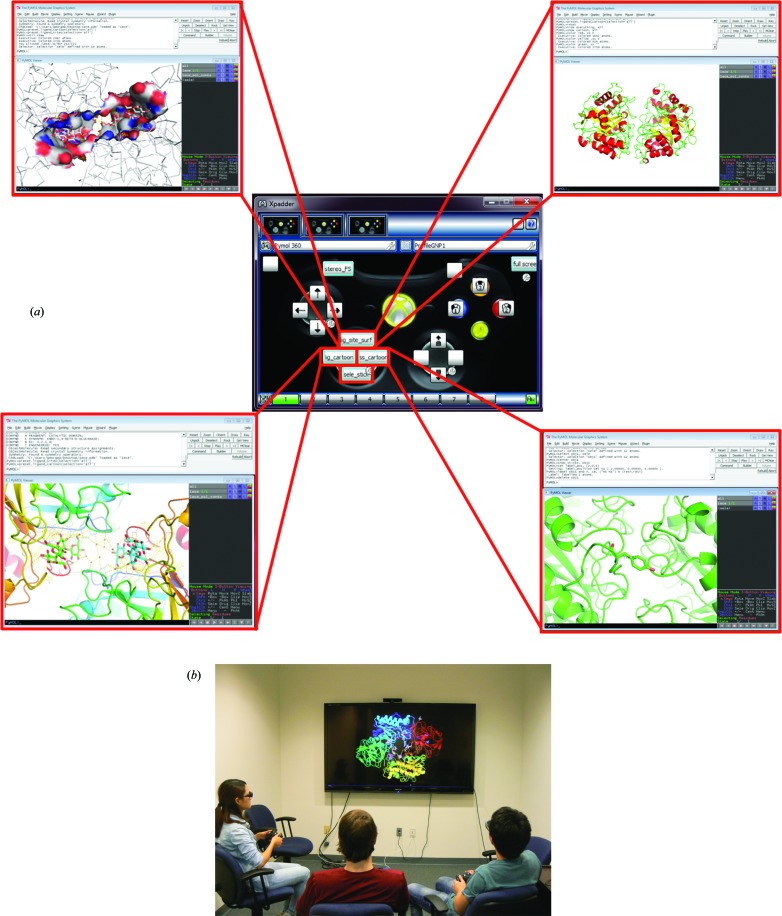
(*a*) *Xpadder* layout for the Xbox controller and the shortcuts implemented. The *Xpadder* layout shows the customized keyboard shortcuts as implemented in *PyMOL*. Mouse functions have been assigned to the left and right stick and the buttons, where X, Y and B are left, middle and right mouse clicks, respectively. Other customized settings for *PyMOL* are as follows: left bumper – stereo and full screen; right trigger – full screen; directional pad (up) – preset of *PyMOL* ‘ligand sites’ and surface colored by atom; directional pad (right) – shows structure as a cartoon and colored by secondary structure; directional pad (down) – selected residue shown as sticks and labeled; directional pad (left) – preset of *PyMOL* ‘ligand cartoon’ showing the polar contacts. (*b*) Current setup of commodity HDTV for co-located discussions. The current setup shows users viewing a protein with stereoscopic glasses and using Xbox game controllers.

Because *Xpadder* maps the keyboard, it becomes possible to execute a set of commands, which would have been typed by users, through a single push of a button. As shown in Fig. 2 ▶, the mouse left, middle and right functions are mapped to the face buttons in the controller. Similarly, the trigger and bumper buttons have been mapped to single commands of *PyMOL*, such as ‘full_screen’ and ‘set stereo on’. Fig. 2 ▶ also shows the directional pad (DP) buttons in the Xbox controller assigned to various keyboard commands. For example, the lower button is labeled as ‘sele_stick’. After a residue in the protein has been selected, the participant can push the button and the residue appears in stick representation, centered and zoomed for detailed view. Similarly, if the protein has a ligand/substrate bound to it, using the DP up or left buttons two different presets can be executed for ligand visualization. The user can show the protein in a cartoon representation by using the right DP button with the classical coloring of helix, strand and loop in red, yellow and green, respectively.

### 4.2. Advantages of this method   

One of the current method’s unique benefits over other existing methods is that the end-user applications (*PyMOL*, *Coot etc.*) do not have to be recompiled or modified. This is beneficial for users who are familiar with applications like *Coot*, *PyMOL*, *VMD* and *Olex2*, as they need not be retrained to use the current setup. Another advantage is that the stereoscopic view enables OpenGL applications with the HDMI 1.4a-based stereoscopic rendering on stereoscopic TV display systems. The user can utilize both active and passive stereoscopic TV display systems.

## 5. Conclusions   

Protein visualization in stereo is extremely valuable for structural biologists. In a structural genomics project structural biologists and their collaborators discuss the structures that have been solved and couple them with the experimental data obtained. Such co-located discussions usually need visualization of the structure and an understanding of the various structural features. In our experience, the study of proteins in an enhanced setting was shown to be helpful in having an effective discussion and also to understand the protein’s structure. Recently, a survey among chemistry and biochemistry educators revealed that visualization of proteins using currently available tools has enhanced their understanding and improved classroom teaching (Craig *et al.*, 2013 ▶). At the same time, new methods of visualization have been developed that have been inspired by the advances in the gaming industry (Lv *et al.*, 2013 ▶). Thus, it is also imperative to develop setups for a co-located collaboration that are easy to recreate without recompiling the visualization software.

Here, we have used inexpensive, commercially available HDTVs with audio-visual converters, requiring no change in the molecular visualization software packages. With the installation of some hardware components, such as the quad-buffer-enabled graphics cards, and installation of the associated drivers, the setup can be easily recreated. The specific elements of the solution may not be very novel. However, by basing the design on an understanding of the task, we were able to tailor the solution and better manage the trade-offs in system functionality, cost and usability. For example, while visualization software would need to be modified, the use of available graphics cards was a trade-off. Similarly, we gave up the three-dimensional volumetric cursor, originally implemented in *CollabMOL*, in exchange for *Xpadder*’s ease of mapping long strings of keyboard command sequences. Together, the elements create a system that demonstrates that a task-informed design can produce an effective collaborative system.
